# Opposing Mitral Valve Clefts, or Perhaps an Isolated Quadricuspid Mitral Valve: A Case Report

**DOI:** 10.7759/cureus.115673

**Published:** 2026-09-02

**Authors:** Scott Heller, Benton Nanners, Klint J Smart, Matthew Lavallee

**Affiliations:** 1 Anesthesiology, West Virginia University, Morgantown, USA; 2 Anesthesiology and Critical Care, West Virginia University, Morgantown, USA

**Keywords:** mitral regurgitation, mitral valve cleft, quadricuspid mitral valve, robotic mitral valve repair, three-dimensional transesophageal echocardiography

## Abstract

A quadricuspid mitral valve is an uncommon congenital anomaly whose imaging features are inconsistently reported. Prior case reports frequently describe this finding in patients with hypertrophic cardiomyopathy (HCM), complicating characterization of this anomaly as an isolated finding.

We report a 64-year-old male with severe mitral regurgitation of incompletely defined origin and atrial fibrillation presenting for robotic thoracoscopic mitral valve repair and a Cox-Maze III procedure. Intraoperative transesophageal echocardiography demonstrated a thickened, myxomatous mitral valve with deep fissures in both the anterior and posterior leaflets extending to the mitral annulus, with the anterior defect being the predominant origin of regurgitation. The opposing mitral valve defects created four apparent leaflet segments, which were suspicious for a quadricuspid mitral valve but ultimately described as a quadricuspid-appearing mitral valve created by opposing true clefts in the anterior and posterior leaflets. No HCM or other structural cardiac anomalies were identified. Surgical repair included direct closure of the anterior leaflet defect, release of two anterior chordae, and placement of a 38-mm annuloplasty band. Post-bypass imaging showed only trivial residual mitral regurgitation. This case highlights the value of three-dimensional transesophageal echocardiography for defining complex mitral leaflet morphology and its importance in surgical planning.

## Introduction

Mitral valve indents, pseudo-clefts, true clefts, and quadricuspid mitral valves are a spectrum of mitral valve anatomy [1]. True clefts and quadricuspid valves likely arise from disruptions during fetal heart development [2]. The most common descriptions and nomenclature suggest that indents extend less than 50% of the leaflet height, pseudo-clefts are greater than 50% but do not reach the mitral annulus, and true clefts are defects that reach the annulus [3,4,5]. Quadricuspid mitral valves have been primarily reported in patients with concomitant hypertrophic cardiomyopathy (HCM) but commonly include four equal-sized mitral leaflets, supernumerary papillary muscles, a square-appearing mitral orifice during diastole, and direct chordal attachments to the newly created commissures [6-9]. We describe a patient whose mitral valve appears quadricuspid; however, several of the previously described characteristics are not present, and therefore the patient's anatomy is best described as having opposing true mitral valve clefts creating a quadricuspid-appearing mitral valve.

Mitral regurgitation can develop as a result of several of the previously described abnormalities, but without clinically significant regurgitation, these subtle defects can be easily missed during a screening transthoracic echocardiogram (TTE). And even with significant regurgitation, which results in referral of a patient to cardiac surgery, the true source may go unrecognized because of the mitral valve’s complex three-dimensional (3D) anatomy. Should a transesophageal echocardiography (TEE) be pursued preoperatively or intraoperatively, clefts have been reported to be difficult to appreciate by two-dimensional (2D) TEE alone and therefore 3D TEE can be particularly helpful in order to define mitral valve morphology and the specific mechanism of regurgitation [10,11]. The patient's anatomy in this case report went unappreciated throughout preoperative workup, including TTE and TEE, but was identified by intraoperative 3D TEE for robotic thoracoscopic mitral valve repair (MVr). This delay in identifying the mechanism of regurgitation did not result in patient harm or change the timeline for patient care, but a detailed description of mitral anatomy intraoperatively did change the surgical plan, resulting in the addition of a direct suture closure of the anterior mitral leaflet cleft.

This case report was previously presented as an abstract at the Society of Cardiovascular Anesthesiologists Annual Meeting on April 27, 2024. Informed consent has been obtained from the patient to publish this case report.

## Case presentation

A 64-year-old man with a past medical history including paroxysmal atrial fibrillation, hypertension, hyperlipidemia, obstructive sleep apnea compliant with a continuous positive airway pressure (CPAP) device, benign prostatic hyperplasia, diet-controlled diabetes mellitus, gastroesophageal reflux disease, moderate-to-severe mitral regurgitation, and heart failure with progressively worsening New York Heart Association (NYHA) III symptoms presented for robotic thoracoscopic MVr and a Cox-Maze III procedure. The patient's preoperative TTE was reported as showing normal biventricular systolic function, severe left atrial dilation (volume index of 89 mL/m²), and a moderately thickened mitral valve with moderate central mitral regurgitation (Carpentier type 1). The patient was then referred for an outpatient TEE with cardiology, which included 3D imaging. The TEE was reported as showing a mildly dilated left ventricle (LV), mildly reduced LV systolic function, a mildly dilated right ventricle (RV), normal RV function, and severe, posteriorly directed mitral regurgitation. The TEE noted a posterior leaflet cleft but made no mention of an anterior leaflet cleft, and no further characterization of the mitral valve was documented.

After induction of general anesthesia, a TEE probe was placed as part of standard perioperative management. 2D TEE showed mild left ventricular dilation (5.5 cm internal cavity diameter), a left ventricular ejection fraction of 55%, and left ventricular wall thickness of 1 cm in the mid-anterior, mid-inferior, and basal septal walls. There was normal right ventricular size and function, no left ventricular outflow tract obstruction by color-flow Doppler, no evidence of systolic anterior motion of the mitral valve, severe left atrial dilation, and a thickened and myxomatous mitral valve with severe, central mitral regurgitation predominating at the A2/P2 region (effective regurgitant orifice area of 0.38 cm², regurgitant volume of 72 mL, and flow reversal in the pulmonary veins). 3D TEE identified both anterior (aortic) and posterior (mural) deep fissures that extended to the mitral annulus, with the predominance of the mitral regurgitation occurring at the central coaptation point of the anterior mitral cleft; there was a slight posterior deviation of the jet flow, which was postulated to be due to orthogonal flow from the anterior cleft (Video 1). A 3D image of the ventricular aspect of the mitral valve demonstrated chordal attachments to the lateral aspect of the anterior cleft (Video 2). These defects produced the appearance of four separate leaflets, consistent with the possibility of a quadricuspid mitral valve (Figure 1). No other structural cardiac anomalies were reported. 

**Video 1:** Philips 3D zoom of the surgeon's en-face mitral valve view with color-flow Doppler. The aortic valve is at the top of the image. The origin of mitral regurgitation predominates at the central coaptation of the anterior mitral cleft.

**Video 2:** The left-side image is the surgeon's en-face mitral valve view, with the aortic valve at the top of the image. There are clefts in both the anterior and posterior leaflets in the A2 and P2 regions, respectively. The right-side image is the ventricular view of the mitral valve, with the aortic valve again at the top of the image. The interatrial septum is to the left of the image, and the lateral wall is to the right. Chordal attachments are appreciated inserting directly into the lateral aspect of the anterior mitral valve cleft.

**Figure 1:**
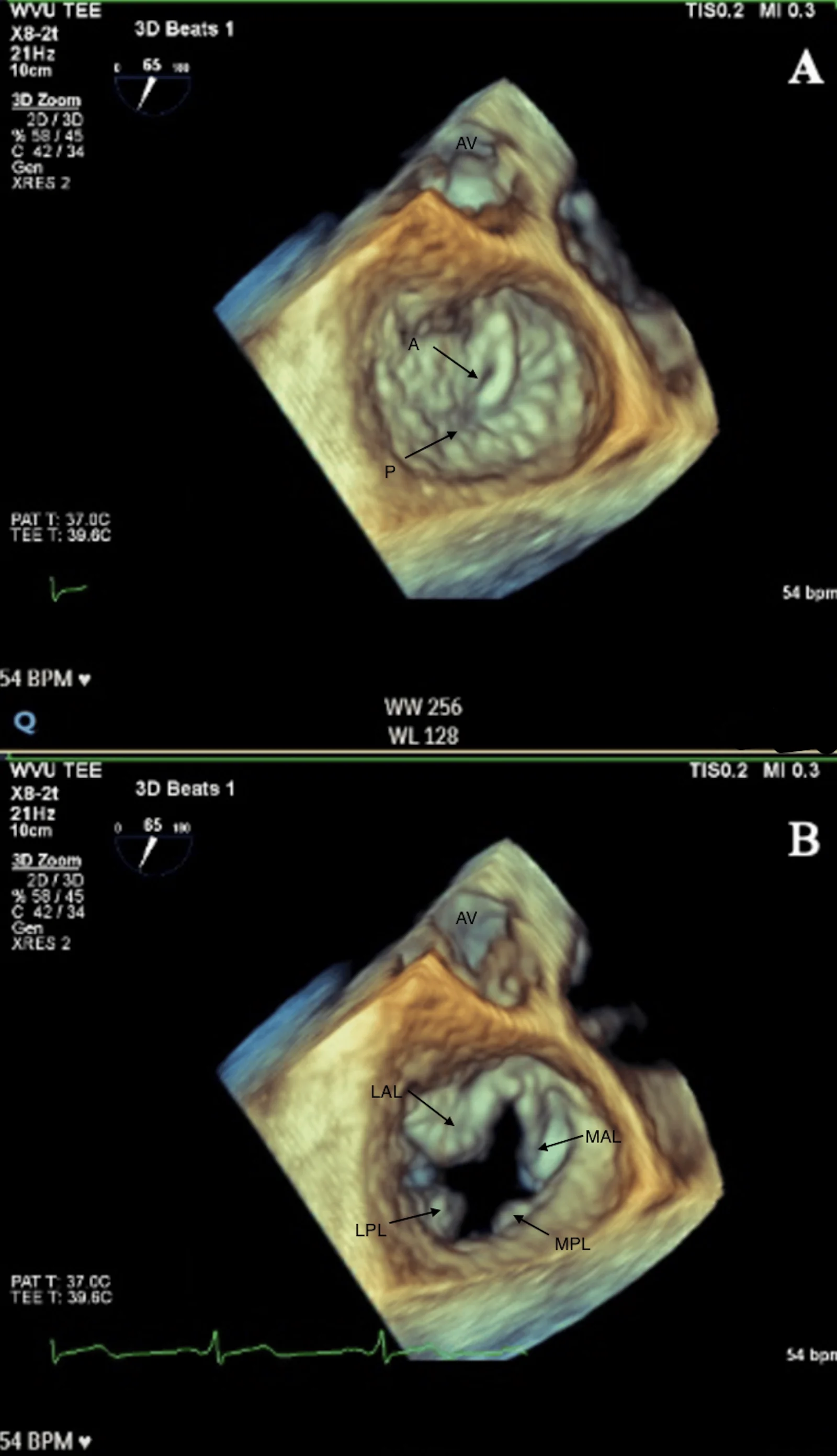
Philips 3D zoom of the mitral valve in systole (A) and diastole (B) from the surgeon's en-face view. Image A demonstrates an anterior cleft (AC) with some prolapse of the coaptation line and a posterior cleft (PC), which is difficult to appreciate. Image B demonstrates the four segments of the mitral valve created by the opposing clefts, labeled with arrows. The aortic valve (AV) is labeled at the top of both images. LAL, lateral segment of the anterior leaflet; MAL, medial segment of the anterior leaflet; LPL, lateral segment of the posterior leaflet; MPL, medial segment of the posterior leaflet

The surgeon noted during direct visualization that there was an anterior cleft reaching the annulus and two chordae inserting into the lateral segment of the anterior leaflet, restricting coaptation. The regurgitation was confirmed to occur predominantly between the anterior leaflet segments created by the anterior cleft. There was no mention of a posterior cleft in the operative report. The valve was repaired by direct suture closure of the anterior leaflet defect, release of two anterior mitral chordae that restricted the cleft from complete coaptation, and implantation of a 38-mm CG Future partial annuloplasty band (Medtronic, Minneapolis, MN). The patient was successfully weaned from cardiopulmonary bypass, with left and right ventricular function reported as normal, only trivial residual mitral regurgitation, and a mean diastolic transmitral valve gradient of 4 mmHg with an atrial and ventricular paced rhythm of 70 beats per minute. The anterior leaflet had full range of motion during diastole, and there was no evidence of incompetence in the region of the previous anterior leaflet cleft. The posterior leaflet was almost completely obliterated from view by the annuloplasty band. The patient's remaining intraoperative and postoperative course was uneventful, with discharge from the hospital on postoperative day 5. At one-month follow-up, the patient was in normal sinus rhythm, and TTE was reported as showing a moderately dilated LV, mildly reduced LV ejection fraction of 42.8%, moderately dilated RV, normal RV function, no evidence of mitral regurgitation, and a mean diastolic transmitral gradient of 4.6 mmHg at a heart rate of 74 beats per minute. The patient continued to endorse shortness of breath with activity at the 30-day outpatient follow-up but subsequently reported no limitations at later follow-up appointments. Only retrospective notes were available to report the patient's symptoms for this case report.

## Discussion

The mitral valve forms from the atrioventricular endocardial cushions in the embryonic heart. These cushions fuse during the sixth week of gestation, dividing the atrioventricular canal into right and left atrioventricular junctions. Normally, the lateral cushion forms the posterior mitral leaflet, while the apposition of the left parts of the superior and inferior cushions forms the anterior leaflet. These transformations create a crescent-like orifice by the eighth week. The two ends of the crescent are connected to compacting columns in the trabecular muscle of the LV, which develop into the anterolateral and posteromedial papillary muscles [3].

Mitral valve indentations, pseudo-clefts, and true clefts creating separate leaflets are likely a spectrum of mitral defects with inconsistent definitions and poor characterization [1]. In the case of true mitral valve clefts and multicuspid mitral valves, normal valve development is likely disrupted, leading to the formation of fissures in the leaflets or apparent separate leaflets [2]. Aberrant endocardial cushion development and fusion, disrupted cell signaling, abnormal remodeling of primitive valve tissue, and genetic factors may all contribute to the development of a quadricuspid mitral valve.

Indentations are naturally occurring in the posterior leaflet of the mitral valve and create the scallops that have been generally accepted as P1, P2, and P3 [12]. However, there are no indentations in the anterior leaflet of the mitral valve. The most commonly proposed definition of an indentation is any defect that extends less than 50% of the height of the leaflet. A defect that extends greater than 50% of the leaflet height but does not reach the annulus is designated as a deep indentation or pseudo-cleft [4,5]. A defect that extends to the annulus is called a true cleft [1,4]. Other distinctions have been proposed, including a functional component whereby concomitant regurgitation from the defect would suggest further subclassification. While the distinction between indentation, pseudo-cleft, and cleft is primarily an interesting academic pursuit, clefts can be a direct contributor to clinically significant regurgitation and require direct closure.

The incidence of cleft or multicuspid mitral valves in the general population is difficult to estimate. There have been a few specific patient populations with reported estimates, including a reported incidence of isolated posterior clefts ranging from 0.11% of all patients referred for TTE in one study to 3.3% of patients with at least moderate mitral regurgitation of unknown origin referred for TEE in a single-center study [2,10]. Many studies have noted that clefts are difficult to identify when using 2D TTE or TEE imaging alone; these defects often require 3D TEE of the mitral valve to be utilized [10,11]. In this patient's case, the anterior leaflet true cleft was never appreciated until the patient arrived in the operating room. While this did not affect the patient's care timeline, identification of the cleft and its contribution to the mitral regurgitation did result in a change in the surgical plan (need for suture closure of the anterior leaflet cleft).

Quadricuspid mitral valves have been previously described as four separate leaflets of similar size, often accompanied by supernumerary papillary muscles. In diastole, the orifice of the mitral valve can take on a square appearance. However, this definition of a quadricuspid mitral valve was created in patients who had concomitant HCM. HCM is correlated with supernumerary papillary muscles, and this also may give rise to the square appearance of the mitral valve [6,7,8]. Therefore, the question arises as to whether these descriptions are specific to quadricuspid mitral valves or influenced by the presence of HCM. In this patient's case, the mitral valve was split into four segments; however, they were not of equal size, and the mitral orifice did not take on a square appearance. To distinguish a cleft from a commissure creating separate leaflets, the presence of primary chordal attachments within the defect has been proposed [9]. There were chordae appreciated inserting into the lateral segment of the anterior leaflet but not the medial segment in this case. These were noted via both echocardiography and the surgeon's direct visualization. Although this patient's mitral valve did appear to be quadricuspid, several of the criteria that have been described previously were not present; therefore, we can only describe this patient's valve as having opposing mitral valve clefts creating a quadricuspid-appearing mitral valve based on current literature.

## Conclusions

Identification and classification of mitral valve indents, clefts, and multicusp valves are convoluted due to imaging difficulties and inconsistent definitions, but with fastidious 3D TEE use, when clinically appropriate, these defects can be appreciated early in a patient's care and an appropriate surgical plan developed to best care for these patients. In this patient, intraoperative 3D TEE provided a detailed assessment of the valve anatomy and clarified the regurgitation mechanism via an anterior cleft that was not fully elucidated preoperatively. Although current literature does not support a diagnosis of a quadricuspid mitral valve in this patient, it is an interesting example of a quadricuspid-appearing mitral valve created by opposing true clefts.
